# Factors Influencing Adoption and Use of Telemedicine Services in Rural Areas of China: Mixed Methods Study

**DOI:** 10.2196/40771

**Published:** 2022-12-23

**Authors:** Yumeng Du, Qiru Zhou, Weibin Cheng, Zhang Zhang, Samantha Hoelzer, Yizhi Liang, Hao Xue, Xiaochen Ma, Sean Sylvia, Junzhang Tian, Weiming Tang

**Affiliations:** 1 Institute for Healthcare Artificial Intelligence Application Guangdong Second Provincial General Hospital Guangzhou China; 2 Internet Hospital Guangdong Second Provincial General Hospital Guangzhou China; 3 School of Data Science City University of Hong Kong Hong Kong China; 4 Gillings School of Global Public Health University of North Carolina at Chapel Hill Chapel Hill, NC United States; 5 Carolina Population Center University of North Carolina at Chapel Hill Chapel Hill, NC United States; 6 University of North Carolina at Chapel Hill Project-China Guangzhou China; 7 Department of Global Health and Population Harvard University Boston, MA United States; 8 Stanford Center for China’s Economy and Institutions Stanford University Stanford, CA United States; 9 China Center for Health Development Studies Peking University Beijing China; 10 Institute for Global Health and Infectious Disease University of North Carolina at Chapel Hill Chapel Hill, NC United States

**Keywords:** telemedicine, telehealth, rural residents, mixed methods, China, mobile phone

## Abstract

**Background:**

The shortage of medical resources in rural China reflects the health inequity in resource-limited settings, whereas telemedicine could provide opportunities to fill this gap. However, evidence of patient acceptance of telemedicine services from low- and middle-income countries is still lacking.

**Objective:**

We aimed to understand the profile of patient end-user telemedicine use and identify factors influencing telemedicine service use in rural China.

**Methods:**

Our study followed a mixed methods approach, with a quantitative cross-sectional survey followed by in-depth semistructured interviews to describe telemedicine use and its associated factors among rural residents in Guangdong Province, China. In the quantitative analysis, explanatory variables included environmental and context factors, household-level factors, individual sociodemographic factors, access to digital health care, and health needs and demand factors. We conducted univariate and multivariate analyses using Firth logistic regression to examine the correlations of telemedicine uptake. A thematic approach was used, guided by the Social Cognitive Theory for the qualitative analysis.

**Results:**

A total of 2101 households were recruited for the quantitative survey. With a mean age of 61.4 (SD 14.41) years, >70% (1364/2101, 72.94%) of the household respondents were male. Less than 1% (14/2101, 0.67%) of the respondents reported experience of using telemedicine. The quantitative results supported that villagers living with family members who had a fever in the past 2 weeks (adjusted odds ratio 6.96, 95% CI 2.20-21.98; *P*=.001) or having smartphones or computers (adjusted odds ratio 3.71, 95% CI 0.64-21.32; *P*=.14) had marginally higher telemedicine uptake, whereas the qualitative results endorse these findings. The results of qualitative interviews (n=27) also supplemented the potential barriers to telemedicine use from the lack of knowledge, trust, demand, low self-efficacy, and sufficient physical and social support.

**Conclusions:**

This study found extremely low use of telemedicine in rural China and identified potential factors affecting telemedicine uptake. The main barriers to telemedicine adoption among rural residents were found, including lack of knowledge, trust, demand as well as low self-efficacy, and insufficient physical and social support. Our study also suggests strategies to facilitate telemedicine engagement in low-resource settings: improving digital literacy and self-efficacy, building trust, and strengthening telemedicine infrastructure support.

## Introduction

### Background

Limited medical resources in rural areas remain a considerable challenge in China, which worsens the health inequity in resource-limited settings [1]. With the shortage of licensed doctors nationwide, there were only 1.56 village doctors and assistants per 1000 rural residents on duty in 2020 [2]. Furthermore, access to quality rural health care services is predicted to degenerate owing to the retirement of current practitioners nearing retirement and emerging opportunities for new health care workers in urban areas [3]. However, booming mobile internet communication and expansion of internet medical services in China suggest a direction for future health care services [4,5]. Telemedicine has the potential to partly fill this huge health care services gap in the rural areas.

As developments of telemedicine in primary care have been boosted since the COVID-19 pandemic crisis [6], the World Health Organization has launched the Global Strategy on Digital Health 2020 to 2025, highlighting the application of digital health technologies for consumers, health professionals, health care providers, and industry to strengthen health systems [7]. The World Health Organization defines telemedicine as “the delivery of health care services using information and communication technologies for the exchange of valid information for diagnosis, treatment and prevention of disease and injuries, research and evaluation, and for the continuing education, all in the interests of advancing the health of individuals and their communities” [8]. Owing to its ability to overcome geographic obstacles to high-quality health care while reducing time and financial costs [5], telemedicine service development is proposed to address medical resource maldistribution in rural areas, especially in the era of the COVID-19 pandemic [9,10]. The high coverage of smartphones and rural telecommunications infrastructure in China have been regarded as favorable conditions for telemedicine accessibility to reach the marginalized and underserved populations [4,11].

### Review of Previous Literature

Understanding the overview of telemedicine use among rural residents is essential for identifying the opportunities and challenges of the adoption of eHealth programs in rural areas. Existing systematic reviews from countries other than China provide evidence regarding factors affecting telemedicine service use from both qualitative and quantitative perspectives [12-14]. Potential barriers such as sociodemographic factors (eg, older age, females, low income, less educated, and physical or mental disability), knowledge or cognitive factors (eg, lack of awareness and information and communication technology skills, lack of demand or motivation, lack of trust in web-based services, and perceived cost), and contextual factors (eg, lack of access to equipment, internet connection, and social support) have been noted [12-14]. However, data on telemedicine or telehealth use from low- and middle-income countries (LMICs) in rural areas were underrepresented [12-14]. In China, most telemedicine programs remained in the pilot stages in metropolitan areas [15,16], and the surveys on telemedicine service use were typically collected from medical professionals or small convenience samples [17,18]. There is a lack of opportunity to study patient end-user acceptance of telemedicine service and its associated factors in rural China.

### Objectives

This study had the following aims: first, to understand the profile of patient end-user telemedicine use among Chinese rural residents, and second, to identify factors influencing telemedicine service use by quantitative and qualitative approaches in rural China.

## Methods

### Study Settings and Design

The prefecture-level cities of Meizhou and Heyuan were selected as study sites. The gross domestic product (GDP) per capita for these cities ranked last and third to last in 2020, respectively, among all cities in Guangdong Province [19]. The GDP per capita of Meizhou City was ¥31,011 (US $4496) in 2020, whereas that of Heyuan was ¥38,802 (US $5625). For comparison, the World Bank classifies economies with per-capita gross national incomes between US $4096 and US $12,695 in 2020 as upper middle income [20]. To address the problem of insufficient primary health care services in rural areas, the Guangdong Provincial Health Commission started to provide smart health monitoring equipment packages to village clinics in 2277 designated “low-income” villages across Guangdong Province in 2019 [21]. The package included telemedicine equipment (including tablets installed with telemedicine software and internet access) and medical devices to conduct examinations (Multimedia Appendix 1). The use of telemedicine services among villagers provided by the Guangdong Provincial Health Commission depends on the patient’s needs and the village doctor’s decision: villagers can request telemedicine consultation when visiting the village clinic with a clear goal of accessing telemedicine service, then the village doctor would conduct remote consultation for the villagers. In addition, the telemedicine provided by Guangdong Provincial Health Commission can serve as an alternative to in-person visits to remote doctors when the village doctor decides to use telemedicine to gain suggestions for patients’ problems. However, villagers can still conduct direct telemedicine consultation on their own through internet-connected devices that they can access (smartphones, computers, etc). The parent project of this study was a village-based cluster randomized controlled trial (CRT)—trial registration number: ChiCTR2100053872—which aimed to increase rural health care use and patient satisfaction, decrease out-of-pocket costs, and improve health outcomes by providing telemedicine platform access and training support to village doctors. Details in treatment and randomization of the CRT can be found in Multimedia Appendix 2.

As part of the baseline research of the CRT in rural areas of Guangdong Province, China, this study followed a mixed methods study approach. A sequential explanatory design was adapted, with a quantitative cross-sectional survey analysis followed by a qualitative thematic analysis of semistructured interviews to identify and explore associated factors of telemedicine use among rural residents.

### Quantitative Approach

#### Sampling and Participant Eligibility

The survey was conducted between July and August 2021. Among all 187 counties in Meizhou and Heyuan cities, 3 counties (Meijiang in Meizhou and Dongxin and Yuangcheng in Heyuan) were first dropped because of a small number of townships. Second, Heping county covering 17 townships in Heyuan was also excluded as a related village doctor training program had recently taken place. Therefore, 167 townships (96 in Meizhou and 71 in Heyuan) were included in the sampling frame. Of these, 144 townships were randomly selected. Villages within these 144 townships would be eligible if (1) they were on the list of 2277 “low-income” villages that were provided smart health equipment packages by the Guangdong Provincial Health Commission; (2) they had at least fifteen households; and (3) the village doctors were willing to receive medical training of the parent CRT. One village was randomly selected from among all eligible villages in the 144 townships. A sampling of 15 households per village was targeted according to health management rosters in each village clinic, including 5 from hypertension and diabetes rosters, 3 with children aged 0 to 6 years and 2 with pregnant or lying-in women. Therefore, a sample of 2160 households were expected. One individual (usually the head or the one most familiar with the household) was suggested by each selected household to respond to the survey. Additional inclusion criteria for respondents of the household survey included (1) a resident in the selected villages in the 2 cities; (2) at least one household member who lived in the village for >3 months in the past year; and (3) willingness to participate in the survey.

#### Data Source and Measurements

The quantitative data were drawn from 2 sources, including the regional economic development data (GDP per capita of each county in 2020) from the government report [22,23] and an interviewer-administered survey questionnaire applied to household respondents.

The binary outcome variable of quantitative data is measured by whether the household’s respondent has ever used the telemedicine platform. To make villagers aware of their experience of telemedicine use, we have explained the term *telemedicine* to villagers by using understandable sentences and phrases. When asking whether they have ever used telemedicine service, we described *using telemedicine* as a process of *seeing a doctor on the web*, *consulting or asking doctors about health issues on the web*, *remotely consulting or asking doctors about health issues through the internet*, or *web-based communications about health issues with the health care professional*. All these situations count as telemedicine use. The explanatory variables included environmental and contextual factors, household-level factors, individual sociodemographic factors, access to digital health care, and health needs and demand factors. The environmental and contextual factors included regional economic factors (GDP per capita of each county in 2020), geographic factors (distance from the village to the town hospital and the most frequently visited hospital), social network factors (having a close relative as a village doctor), and health system factors (measured by the number of house calls by village doctors in the past year). At the household level, the family size (number of family members), financial situation of the household (wealth index calculated by principal component analysis of household assets), and whether the family is in the poverty registration were included. Individual sociodemographic factors included whether they are the head of the household, age, sex education, and participation in insurance. Access to telemedicine services, smartphone or computer ownership, and others’ help using the internet were investigated. Health needs and demand factors of family members include the prevalence of chronic disease (hypertension and diabetes), acute disease and symptoms (diarrhea, cough, or runny nose in the past year, and fever in the past 2 weeks), and health-seeking behaviors (frequency of visits to the village clinic, township hospital, and county hospital).

#### Quantitative Analysis

The quantitative data were exported to Stata SE 16.0 (StataCorp) for statistical analysis. Descriptive statistics such as frequencies (n), proportions (%) with 95% CIs, and numerical summary measures (including means and SDs) were used to describe the data. Cluster adjustment for SE was used in CI estimation of characteristic proportions to modify the estimation in our cluster-sampling observational study. Variance inflation factor was calculated to assess multicollinearity between variables [24]. Age and gender were defined as prior confounder and forced variables that entered the regression model. To address data *separation* and minimize bias caused by conventional logistic model maximum likelihood estimation resulted from the extremely low rate of telemedicine use in this study [25,26], Firth logistic regression that was proposed as an ideal strategy for rare events in 2020 SAS Global Forum based on penalized likelihood strategy was adapted [27]. Both univariate and multivariate analyses were conducted using regression models presented as crude and adjusted odds ratios (aORs) with 95% CIs. The coefficient of discrimination D (also known as Tjur *R*^2^) was calculated to indicate goodness-of-fit [28]. The receiver operating characteristic of the area under the curve (ROCAUC) was adapted to evaluate the predictive power of the Firth logistic regression [29]. The level of statistical significance was declared at *P* values of <.05.

### Qualitative Approach

#### Sampling and Participant Eligibility

Semistructured face-to-face interviews were conducted for qualitative research between January and February 2022. A total of 8 local interviewers who could communicate with villagers in a local accent (ie, Hakka) and lived in 8 rural villages were recruited. The list of the 8 villages can be found in Multimedia Appendix 3. To gain a comprehensive understanding of barriers to and facilitators of telemedicine use among different groups of people through qualitative study, a purposive sampling strategy was used during interviewee selection [30]. For key topics of behavioral factors (eg, experience and practice) in the Social Cognitive Theory (SCT) would mainly be explored among telemedicine users and extremely low telemedicine use (14/2101, 0.67%) informed by quantitative survey, we purposefully sampled participants with and without experience of telemedicine use. Each interviewer was fully trained before they conducted the interviews. The interviewees’ eligibility for qualitative interviews was similar to that of the quantitative survey: (1) lived in the village in Meizhou or Heyuan for >3 months in the past year; (2) lived with family members in the roster of hypertension, diabetes, pregnant or lying-in women, or children aged 0 to 6 years from the village clinic; and (3) agreed to participate in the interview.

#### Conceptual Framework and Data Collection

**Figure 1:**
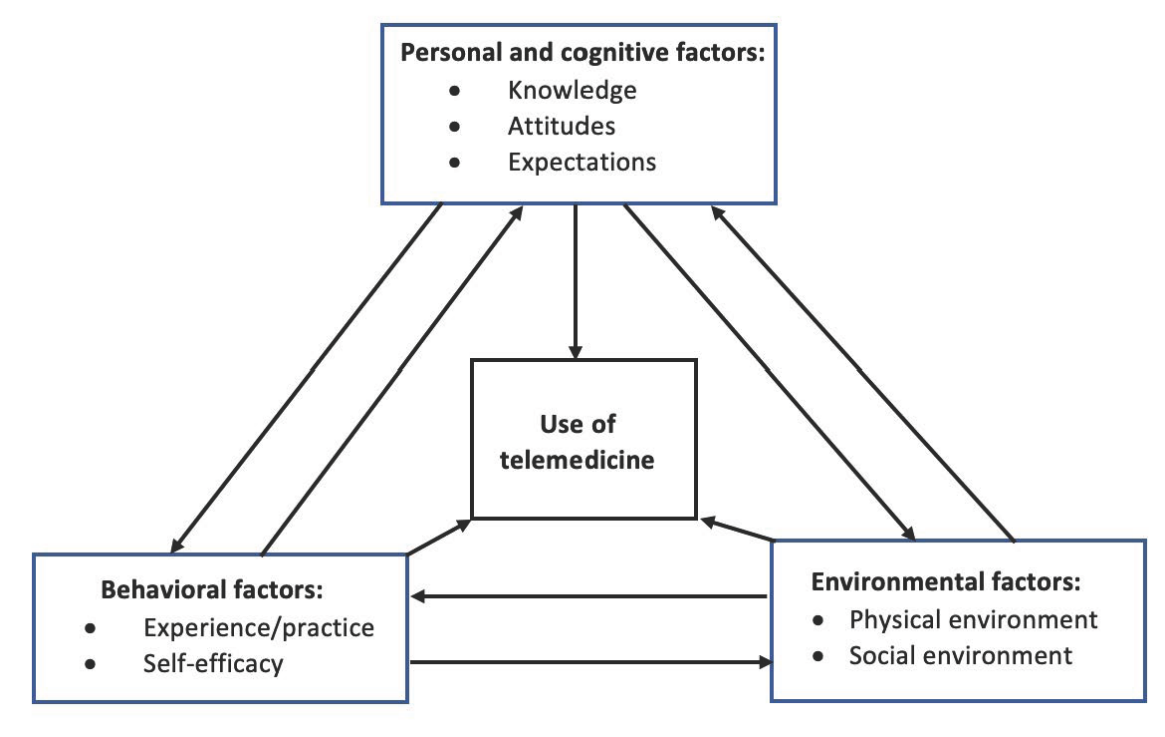
Adapted social cognitive model.

Topic guides were developed for the semistructured in-depth interviews based on SCT and an extensive review of the existing literature [12-14]. SCT is a framework that includes 3 main components that interact with each other bidirectionally: cognition and personal factors, behavior factors, and environmental factors [31]. The conceptual framework using the adapted SCT is shown in Figure 1. Considering that SCT has been used in research on health behavior and information system adoption behavior [32,33] and helped to obtain a comprehensive understanding of the mechanism that affected behavioral intentions to use telemedicine, SCT would be an appropriate theoretical basis to guide future studies. The key topics of each SCT main component were developed according to the previous research (Table 1) [12-14]. From the cognitive perspective, personal understanding and knowledge, attitudes and perceptions, and expectations related to telemedicine were asked. Regarding behavioral factors, topics focused on self-efficacy, asking about how confident the respondents thought they were able to use telemedicine to meet their health-related demands. Experience and past practice was also asked of those who already had a history of telemedicine use. Both physical and social environmental factors affecting the use of telemedicine services were included. The specific interview questions for each key topic can be found in Multimedia Appendix 4.

**Table 1:** Operational definitions and key topics examined for the Social Cognitive Theory (SCT) components explored during in-depth interviews with rural residence in Guangdong Province, China.

SCT components and operational definition				Key topics explored
**Cognitive factors**				
	Knowledge: knowledge to perform telemedicine consultation		Information source, awareness, and operation	
	Attitude: positive and negative attitude toward telemedicine service		Necessity, confidence, cost, convenience, benefit, and risk	
	Expectation: expectations for telehealth services and health outcomes		Expected assist, service function, and health goal	
**Behavior factors**				
	Experience and practice: experience and past practice of telemedicine use		Difficulty, improvement, and doctor assessment	
	Self-efficacy: the extent to which the respondents believe they were able to use telemedicine to meet their health-related demands		Ability to access information or operate device or communicate on the web or seek assistance	
**Environmental factors**				
	Physical support: equipment and facility regarding telemedicine service		Internet connection, smartphone, and computer	
	Social support: social support that may facilitate telemedicine use		Training, financial reimbursement, professional recommendation, and external assist	

The interview is based on a semistructured interview guide based on SCT. Communications were conducted in Mandarin or Hakka (for villagers who could not speak or understand Mandarin). Oral consent was sought and audio recorded before the formal interview. Anonymity and confidentiality were stressed. We continued the interview until data saturation, which refers to the stage when the interviewer keeps hearing repeated information or the interviewee cannot provide new information during interview data collection, was reached [34]. Each interview was also fully audio recorded and transcribed. Interviews that were conducted in Hakka were translated into Mandarin for transcription. One of the authors verified the accuracy of the transcripts.

#### Qualitative Analysis

Data were analyzed using a thematic approach with the assistance of NVivo (version 12). An inductive coding process was conducted and a codebook was set up. A total of 2 researchers independently read and coded text materials and double-coded interview transcripts using a line-by-line, open-coding process to check for consistency and accuracy. Divergences were then discussed until a consensus was reached. Afterward, codes were categorized into different themes or subthemes according to the adapted SCT model (Figure 1). Quotes were translated from Chinese to English for the write-up.

### Ethical Considerations

We obtained multicenter ethics approval from Guangdong Second Provincial General Hospital (Institutional Review Board [IRB] approval number: GD2H-KY IRB-AF-SC.07-01.1), Peking University (IRB approval number: IRB00001052-21007-免), and the University of North Carolina at Chapel Hill (IRB approval number: 21-0549). It has also been registered in the clinical trial registry in China (ChiCTR2100053872). Written informed consent was obtained from all participants in the quantitative survey. Oral informed consent was obtained and recorded from the participants for qualitative interviews. Confidentiality and privacy was assured for every respondent in the survey and the interviews. All stored data were encrypted in computer-based files, and any information used for research purposes was deidentified. Data stored in computer-based files are only accessible to those with proper clearance. Only study members can access identifiable data. All survey participants received a towel worth ¥15 (US $2) as compensation for their time and inconvenience of participation in the research.

## Results

### Quantitative Findings

#### Descriptive Analysis

**Figure 2:**
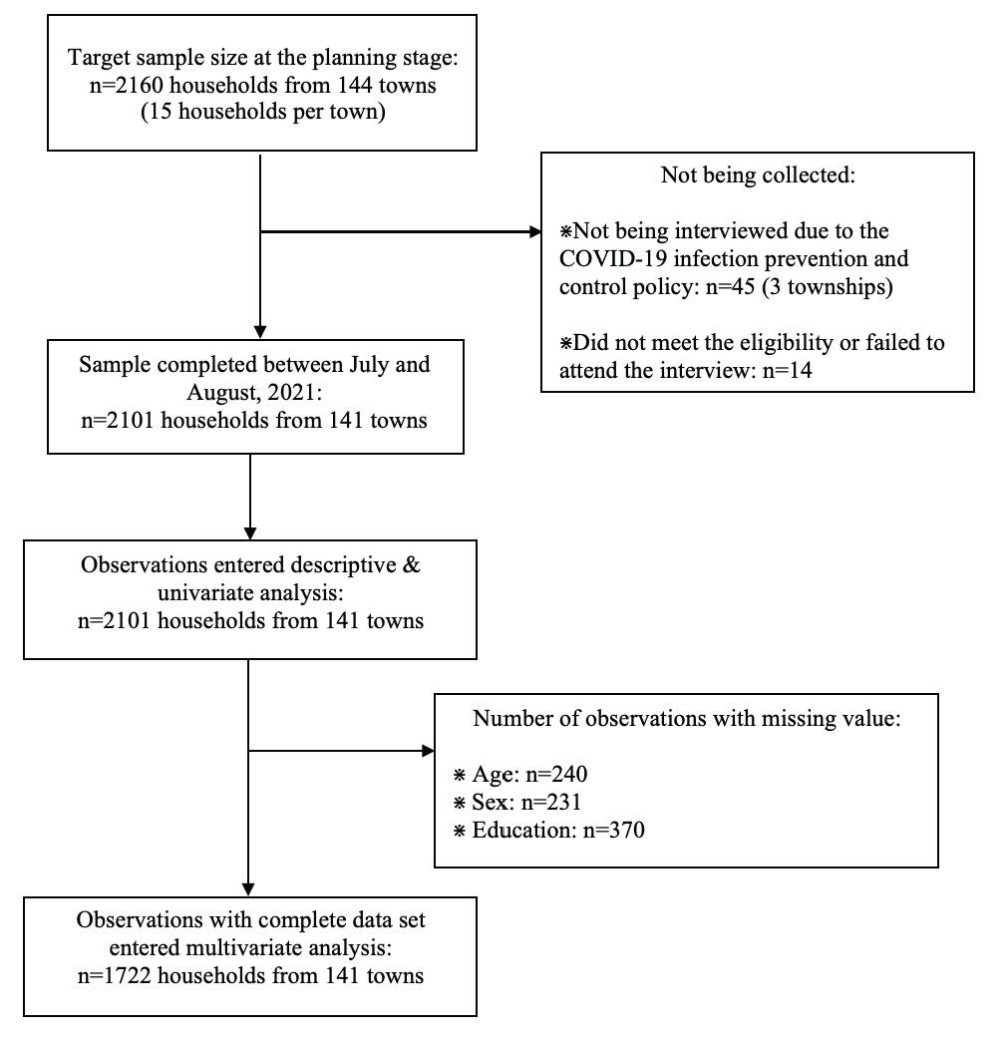
STROBE (Strengthening the Reporting of Observational Studies in Epidemiology) diagram for progress through stages of analyses.

During the baseline investigation, 2101 households and 141 village clinics were examined. Figure 2 shows a diagram of the Strengthening the Reporting of Observational Studies in Epidemiology presenting the stages of the analytic process. We were unable to reach 45 households from 3 village clinics (villages) because of the SARS-CoV-2 infection prevention and control policy implemented during the investigation. In addition, 14 households were excluded for not meeting the eligibility criteria or failing to participate in the interview. Among the information from 2101 households being collected, 379 individuals with missing values of age, gender, or education of the main respondent did not enter multivariate analysis but were included in the descriptive and univariate analyses.

Among all the respondents (N=2101), only 14 (0.67%) reported having ever used telemedicine services. Among participants who had used telemedicine services before (n=14), 3 (21%) of them reported having registered for medical services on the web and 2 (14%) of them reported having a web-based service for doctors’ follow-up visits.

Table 2 presents the characteristics of the participants (N=2101) categorized by various factors: environment and context, household, individual sociodemographic, access, and health needs and demand. With a mean age of 61.4 (SD 14.41) years; >70% (1364/2101, 72.94%) of the respondents were male. More than 80% of the respondents were the heads of households. More than half (1021/2101, 58.98%) of the participants had a degree from senior high school or technical secondary school. In terms of access to digital health care, 67.06% (1409/2101) of participants reported ownership of a smartphone or computer, 62.54% (1314/2101) had internet connection, and 37.65% (791/2101) said they had someone who could help them use the internet in their family. Regarding health needs and demands of family members, hypertension (1405/2101, 66.87%) was the most reported disease, whereas fever in the past 2 weeks (134/2101, 6.38%) was the least reported.

**Table 2:** Characteristics of rural residents in Guangdong Province, China (N=2101).

Characteristic and category				Observations		95% CI^a^ of the percentage or mean
**Environmental and contextual factor, n (%)**						
	**Per capital GDP^b^ of each county in 2020 (Chinese yuan renminbi)**					
		<25,000 (US $3623)	986 (46.93)		38.74-55.29	
		≥25,000 (US $3623)	1115 (53.07)		44.71-61.26	
	**Distance from village to the town hospital (km)**					
		<5.0	1029 (48.78)		40.74-57.27	
		≥5.0	1072 (51.02)		42.73-59.26	
	**Distance from village to the most frequently visited county hospital** **(km)**					
		<35.0	1026 (48.83)		40.58-57.15	
		≥35.0	1075 (51.17)		42.85-59.42	
	**Being close relative of the village doctor**					
		No	1964 (93.48)		92.14-94.60	
		Yes	137 (6.52)		5.40-7.86	
	**Number house calls by village doctors in the past year**					
		None	982 (46.74)		42.48-51.05	
		1-3	484 (23.04)		20.77-25.48	
		≥4	635 (30.22)		26.74-33.95	
**Household-level factor, n (%)**						
	**Family size (number of family members)**					
		1	203 (9.66)		8.34-11.16	
		2	545 (25.94)		23.93-28.05	
		3-5	626 (29.80)		27.73-31.94	
		>5	727 (34.60)		32.05-37.25	
	**Financial situation (wealth index)**					
		<20%	420 (19.99)		17.66-22.54	
		20%-40%	411 (19.56)		17.85-21.39	
		40%-60%	429 (20.42)		18.64-22.32	
		60%-80%	419 (19.94)		18.16-21.85	
		≥80%	422 (20.09)		17.93-22.43	
	**Family in poverty registration**					
		No	1735 (82.66)		80.76-84.41	
		Yes	364 (17.34)		15.59-19.24	
**Individual sociodemographic factor**						
	**Householder, n (%)**					
		No	288 (15.40)		13.37-17.68	
		Yes	1582 (84.60)		82.32-86.63	
	**Age (years), mean (SD)**					
		Continuous	61.4 (14.41)		60.57-62.15	
	**Sex, n (%)**					
		Male	1364 (72.94)		70.46-75.29	
		Female	506 (27.06)		24.71-29.54	
	**Education level, n (%)**					
		Junior high school or below	710 (41.02)		38.09-44.00	
		Senior high school or above	1021 (58.98)		56.00-61.91	
	**Use of any medical insurance programs** **, n (%)**					
		No	89 (4.24)		3.32-5.38	
		Yes	2012 (95.76)		94.62-96.67	
**Access to digital health care, n (%)**						
	**Having smartphone or computer**					
		No	692 (32.94)		30.73-35.22	
		Yes	1409 (67.06)		64.78-69.27	
	**Can be connected to the internet**					
		No	95 (37.46)		35.15-39.83	
		Yes	1314 (62.54)		60.17-64.85	
	**Someone can help to use the internet in the family**					
		No	1310 (62.35)		59.80-64.83	
		Yes	791 (37.65)		35.17-40.20	
**Health needs and demand of family members, n (%)**						
	**Hypertension**					
		No	696 (33.13)		31.27-35.03	
		Yes	1405 (66.87)		64.97-68.73	
	**Diabetes**					
		No	1236 (58.83)		56.98-60.65	
		Yes	856 (41.17)		39.35-43.02	
	**Diarrhea in the past year**					
		No	1249 (59.48)		56.65-62.19	
		Yes	852 (40.55)		37.81-43.34	
	**Cough or runny nose in the past year**					
		No	905 (43.07)		40.76-45.42	
		Yes	1196 (56.93)		54.58-59.24	
	**Fever in the past 2 weeks**					
		No	1967 (93.62)		92.39-94.67	
		Yes	134 (6.38)		5.33-7.61	
	**Frequency of the county hospital visit in the past year**					
		None	1328 (63.21)		60.61-65.74	
		1 time	343 (16.33)		14.76-18.02	
		≥2 times	430 (20.47)		18.51-22.5	
	**Frequency of the town hospital visit in the past year**					
		None	1028 (48.93)		46.02-51.85	
		1 time	274 (13.04)		11.48-14.78	
		≥2 times	799 (38.03)		35.19-40.95	
	**Frequency of the village clinic visit in the past year**					
		None	893 (42.50)		38.79-46.31	
		1-3 times	318 (15.14)		13.61-16.80	
		≥3 times	890 (42.36)		39.02-45.78	

^a^CI of the percentage estimation was adjusted by SE of cluster sampling.

^b^GDP: gross domestic product.

#### Correlation of Telemedicine Use

Table 3 shows the results of the crude and aORs in using telemedicine services with a 95% CI. In univariate analysis, participants who had family members who developed a fever in the past 2 weeks (OR 6.42, 95% CI 2.10-19.69; *P*=.001) were more likely to use telemedicine services than those who did not. In addition, smartphone or computer ownership (OR 1.47, 95% CI −0.31 to 3.25; *P*=.08) also tended to use telemedicine compared with their counterparts. Participants living with family members who had hypertension (OR 0.38, 95% CI 0.14-1.05; *P*=.06) or fever in the past 2 weeks (OR 6.42, 95% CI 2.10-19.69; *P*=.001) were less likely to accept telemedicine services. However, after adjusting for age and gender, only people with family members who developed a fever in the past 2 weeks (aOR 6.96, 95% CI 2.20-21.98; *P*=.001) had significantly higher uptake rates of telemedicine services. Evidence regarding the association between other independent factors and telemedicine uptake is insufficient. The variance inflation factor of each explanatory variable shown in Multimedia Appendix 5 signifies the absence of multicollinearity in the model. Multimedia Appendix 6 shows Tjur *R*^2^ and ROCAUC for each multivariate model. The multivariate model including independent variables of fever history in the past 2 weeks, age, and gender indicated that >58% of the variance for telemedicine use was explained by independent variables (Tjur *R*^2^=0.582) and fair predictive power of the fitted model (ROCAUC=0.718).

**Table 3:** Firth logistic regression analysis of correlation of telemedicine use for rural residents in Guangdong Province, China (N=2101).

Characteristic and category				Crude OR^a^ (95% CI)		*P* value^b^		aOR^c^ (95% CI)^d^		*P* value^e^
**Macrocontext factor**										
	**Per-capita GDP^f^ of each county (Chinese yuan renminbi)**									
		<25,000 (US $3623)	Ref^g^		.78		N/A^h^		.92	
		≥25,000 (US $3623)	1.16 (0.41-3.22)		.78		0.95 (0.33-2.75)		.92	
	**Distance from village to the town hospital (km)**									
		<5.0	Ref		.94		N/A		.80	
		≥5.0	0.96 (0.35-2.64)		.94		1.13 (0.40-3.29)		.80	
	**Distance from village to the most frequently visited county hospital (km)**									
		<35.0	Ref		.27		N/A		.15	
		≥35.0	0.55 (0.19-1.58)		.27		0.43 (0.14-1.34)		.15	
	**Being close relative of the village doctor**									
		No	Ref		.62		N/A		.63	
		Yes	0.49 (0.03-8.25)		.62		0.50 (0.03-8.41)		.63	
	**Number house calls by village doctors in the past year**									
		None	Ref		.27		N/A		.20	
		1-3	1.58 (0.39-6.42)		.27		1.12 (0.24-5.31)		.20	
		≥4	2.59 (0.80-8.41)		.27		2.69 (0.83-8.76)		.20	
**Household-level factor**										
	**Family size (number of family members)**									
		1	Ref		.47		N/A		.76	
		2	2.64 (0.14-50.91)		. 47		2.36 (0.12-46.06)		.76	
		3-5	2.29 (0.12-44.27)		. 47		2.01 (0.10-39.65)		.76	
		>5	4.81 (0.28-83.93)		. 47		3.42 (0.19-62.80)		.76	
	**Financial situation (wealth index)**									
		<20%	Ref		.09		N/A		.11	
		20%-40%	4.48 (0.76-26.56)		.09		4.44 (0.74-26.31)		.11	
		40%-60%	0.33 (0.01-8.00)		.09		0.29 (0.01-7.17)		.11	
		60%-80%	1.00 (0.10-9.68)		.09		0.21 (0.09-8.33)		.11	
		≥80%	4.34 (0.73-25.79)		.09		3.06 (0.49-19.30)		.11	
	**Family in poverty registration**									
		No	Ref		.54		N/A		.42	
		Yes	1.45 (0.44-4.81)		.54		1.63 (0.49-5.53)		.42	
**Individual sociodemographic factor**										
	**Householder**									
		No	Ref		.80		N/A		.86	
		Yes	0.84 (0.21-3.32)		.80		5.53 (0.18-7.69)		.86	
	**Age (years)**									
		Continuous	0.98 (0.95-0.99)		.16		0.98 (0.25-1.01)		.15	
	**Sex**									
		Male	Ref		.86		N/A		.80	
		Female	0.90 (0.27-3.03)		.86		0.85 (0.25-2.89)		.80	
	**Education level**									
		Junior high school or below	Ref		.50		N/A		.73	
		Senior high school or above	1.48 (0.48-4.53)		.50		1.23 (0.38-4.01)		.73	
	**Use of any medical insurance programs**									
		No	Ref		.29		N/A		.27	
		Yes	0.40 (0.07-2.18)		.29		2.64 (0.07-2.10)		.27	
**Access to digital health care**										
	**Having smartphone or computer**									
		No	Ref		.08		N/A		.14	
		Yes	4.43 (0.83-24.05)		.08		3.71 (0.64-21.32)		.14	
	**Can be connected to the internet**									
		No	Ref		.13		N/A		.22	
		Yes	2.89 (0.73-11.36)		.13		2.46 (0.58-10.38)		.22	
	**Someone can help to use the internet in the family**									
		No	Ref		.65		N/A		.56	
		Yes	1.27 (0.45-3.53)		.65		1.36 (0.48-3.90)		.56	
**Health needs and demand of family members**										
	**Hypertension**									
		No	Ref		.06		N/A		.17	
		Yes	0.38 (0.14-1.05)		.06		0.46 (0.15-1.40)		.17	
	**Diabetes**									
		No	Ref		.38		N/A		.26	
		Yes	0.61 (0.20-1.82)		.38		0.49 (0.14-1.68)		.26	
	**Diarrhea in the past year**									
		No	Ref		.46		N/A		.36	
		Yes	1.46 (0.53-4.06)		.46		1.63 (0.57-4.71)		.36	
	**Cough or runny nose in the past year**									
		No	Ref		.59		N/A		.46	
		Yes	0.76 (0.27-2.10)		.59		0.67 (0.23-1.92)		.46	
	**Fever in the past 2 weeks**									
		No	Ref		.001		N/A		.001	
		Yes	6.42 (2.10-19.69)		.001		6.96 (2.20-21.98)		.001	
	**Frequency of the county hospital visit in the past year**									
		None	Ref		.34		N/A		.60	
		Once	0.68 (0.12-3.86)		.34		0.70 (0.12-4.01)		.60	
		≥Twice	1.52 (0.49-4.76)		.34		1.62 (0.51-5.10)		.60	
	**Frequency of the town hospital visit in the past year**									
		None	Ref		.44		N/A		.48	
		Once	2.41 (0.63-9.21)		.44		2.27 (0.59-8.76)		.48	
		≥Twice	N/A		.44		1.26 (0.38-4.14)		.48	
	**Frequency of the village clinic visit in the past year**									
		None	Ref		.29		N/A		.41	
		1-3 times	0.15 (0.01-2.51)		.29		0.17 (0.01-2.89)		.41	
		≥3 times	0.57 (0.20-1.67)		.29		0.68 (0.23-2.01)		.41	

^a^OR: odds ratio.

^b^*P* value for penalized likelihood-ratio test of univariate model.

^c^aOR: adjusted odds ratio.

^d^Adjusted odds ratio: marginal effect adjusted for age and sex in the multivariate logistic model.

^e^*P* value for likelihood-ratio test of multivariate model.

^f^GDP: gross domestic product.

^g^Ref: Reference value.

^h^N/A: not applicable.

### Qualitative Findings

#### Overview

A total of 27 semistructured individual interviews were conducted with villagers from 8 villages. Background characteristic numbers (P1-P27) of each interviewee are shown in Multimedia Appendix 3. The average time length of 27 interviews was 25.6 (SD 11.2) minutes. The mean age of the interviewees was 53.4 (SD 16.7) years, and 56% (15/27) were male. Of these, 22% (6/27) said they had never heard of telemedicine services before, and 44% (12/27) said they had heard of telemedicine but never used it. Of all the interviewees, 33% (9/27) reported having experience with telemedicine use. A total of 10 themes, classified into 3 categories (personal and cognitive, behavioral, and environmental factors), were extracted from the interview content according to the adapted SCT (Table 4). Major themes that emerged as barriers to telemedicine service uptake among villagers included lack of knowledge and understanding, lack of trust, lack of demand, low self-efficacy, lack of physical support, and social support.

**Table 4:** Themes and subthemes of qualitative analysis on telemedicine use among rural residence in Guangdong Province, China (N=27).

Theme and subtheme				People mentioned, n (%)		Coding
**Personal and cognitive factors**						
	**Knowledge and understanding**					
		Web-based process recognized	16 (59)		Web-based searching, appointment, registration, consultation, diagnosis, prescription, and payment	
		Operation	18 (67)		Lack of technical ability and lack of comprehension ability	
	**Attitude**					
		Perceived convenience	27 (100)		Avoid queuing up, avoid COVID-19 prevention policy limit, and overcome traffic barrier and distance	
		Perceived cost	25 (93)		Financial cost, time cost and efficiency, and manpower	
		Lack of trust	19 (70)		Concern about fraud or false information, personal information security, government authority and supervision, doctor’s certificate, doctor’s seniority, and reputation of the hospital	
		Lack of demand	27 (100)		Complacent about self-condition, local clinic and doctor’s indoor visit as alternatives, auxiliary and reference for offline visit, chronic disease, minor aliment, and rush hour or weekends	
	**Expectation**					
		Health expectation	16 (59)		Chronic disease management and address minor disease	
		Service expectation	21 (78)		Guide for healthy lifestyle and knowledge promotion	
**Behavioral factors**						
	**Experience and practice**					
		Web-based process experience	6 (22)		Appointment, registration, prescription, and taking photos for doctors on the web	
		Difficulty or improvements	5 (19)		Web-based communications, lack of information interoperability and integrality across platforms	
		Assessment of the doctor	8 (30)		Web-based feedback, doctor’s title, and doctor’s resume,	
	**Self-efficacy**		27 (100)		Web illiterate, aging, memory loss, dull-witted, illiteracy, visually impaired, dialect barriers, and out of step with the times	
**Environmental factors**						
	**Lack of physical support**					
		Equipment	11 (41)		Internet connection and smartphone or computer devices	
		Technical limitations	13 (48)		Lack of physical examination, lack of physiological tests, lack of medical imaging tests, and low video or photo resolution	
		Delivery system	10 (37)		Lack of direct home delivery service, lack of express pickup points in the village, and delay of drug delivery	
	**Social support**					
		Training support	21 (78)		Desire for teaching and training in the village	
		Role of primary health care provider	25 (93)		Improve ability of village doctors, provide assistance for villagers, and formalists	

#### Themes and Quotes

##### Lack of Knowledge and Understanding

The knowledge theme indicated limited understanding and knowledge of telemedicine among the interviewees. Most (18/27, 67%) of the respondents said they did not know how to use or were not good at operating telemedicine services. However, some provided examples of the web-based process (eg, appointment, register, consultation, and prescription):

We basically don’t know how to use smartphones. I can't even take a taxi with a mobile phone.

P27, female, 27 years

##### Lack of Trust

Many (10/19, 53%) villagers showed negative perceptions about reliability, concern about internet scammers, and personal information leakage of the telemedicine technology. In addition, some (5/19, 26%) demonstrated conditional trust, meaning that they would trust telemedicine services if conditions such as doctors’ certificates, supervision or regulations of government, and reputations of doctors and hospitals were in place:

Now there are so many scammers, the Internet has the most of them. If you can't find reliable one, I'm afraid no one will take the risk for you. Does Alipay have a platform called Huzhubao? Is such a big platform gone now?

P20, female, 25 years

There is such a possibility, I am not so relieved. If the doctor does something bad using our ID card information, we will not be able to figure it out. People are unpredictable, especially those who have only met once.

P3, female, 25 years

I believe that the Internet could be reliable to a certain extent. It should have a certificate issued by the nation, involving a relatively well-known hospital or doctors, etc.

P7, female, 48 years

##### Lack of Demand

Many (15/27, 56%) responders did not think telemedicine was a necessity. Several (4/27, 15%) villagers said that they were complacent about their health condition or would prioritize physical hospitals (7/27, 26%). Some participants (14/27, 52%) mentioned that they would use telemedicine only if they encountered a *minor ailmen*t (eg, cold or fever). This finding was consistent with our quantitative findings that showed higher telemedicine service uptake among villagers who had family members with fever in the past 2 weeks (aOR 6.96, 95% CI 2.20-21.98; *P*=.001). Our qualitative findings also indicated that offline hospitals were preferred when dealing with a *serious disease*; respondents tended to regard the web-based results as a reference for seriousness:

It is not necessary for me (to use a telemedicine platform). I'm in good health now.

Maybe for the time being, doctors on the Internet are still relatively unfamiliar to me. There is no need to use this approach. We all can go to the local hospital for treatment. My idea is that if we encounter a problem that the local doctor can’t solve, we could then take this approach (telemedicine).

P22, male, 60 years

It depends, some simple disease such as cold or fever, I would go online for consultation. It’s OK to just have a look (in an Internet hospital). If it’s a serious illness, I won’t go online. I will only regard the online results as a reference.

P11, male, 29 years

##### Low Self-efficacy

Most (16/27, 59%) of the participants expressed negatively about self-efficacy, for they thought themselves as aging, illiterate, dull, and forgetful people and did not believe they had the ability to use telemedicine:

We elderly have never used it. We are illiterate, have never been on the Internet. We can't surf the Internet, our eyes are not very good, and we can't read a few words.

P25, male, 68 years

I'm old, with limited operational abilities, dullness, and memory loss.

P15, male, 72 years

##### Lack of Physical Support

The lack of smartphone or computer or internet connection could be a barrier to telemedicine adoption in our interviews. This was also supported by quantitative findings suggesting that smartphone or computer ownership (aOR 3.71, 95% CI 0.64-21.32; *P*=.14) among villagers was marginally correlated with telemedicine uptake:

My old-fashioned cell phone does not have such functions. Some middle-aged people can accept it. Of course, for young people it’s needless to say. It (internet platform) is very popular among them.

P22, male, 60 years

Besides equipment, technical limitations (13/27, 48%) and delivery systems (10/27, 37%) were also identified as insufficient physical support. Concerns about technical limitations mainly come from a lack of medical examination instruments or physical contact with doctors. Interviewees did not think doctors could make an accurate diagnosis by simply asking on the web or uploading photos without any laboratory tests or feeling the pulse (traditional Chinese medicine). Issues with the delivery system were said to cause inconvenience for drug delivery (10/27, 37%); a pharmacy pickup point located in the village or rapid home delivery was needed:

Laboratory tests are also required in the hospital, if the doctor purely questions the patient, it may not be so accurate. In traditional Chinese medicine, seeing a doctor in person is like seeing, hearing, asking, and feeling the pulse. That is definitely not so good if you visit the doctor online

P4, female, 46 years

If there is no pharmacy in the village for Internet hospitals, and I have to wait for 3-4 days for the medicine, and then go to the town or county to get the medicine, then I would rather go to the village doctor. It is not very cost-effective to see a doctor online and wait for three or four days for the medicine.

P3, male, 60 years

##### Social Support

The social environment described the social interaction with the villagers that may influence their telemedicine use, including training support and the roles of primary health care providers. Most (19/21, 90%) people expressed their desire or positive view of training support for telemedicine:

I may need to see a doctor online in the future, but only if someone teaches me. If someone teaches me, I will be more willing to go to see a doctor online.

P16, male, 53 years

Most (14/25, 56%) interviewees held positive views on the use of telemedicine among primary health care providers. They also thought that primary health care providers could improve their abilities and skills by using telemedicine and help to guide and oversee the process of seeking medical treatment from a superior hospital:

It is good (when village doctors using telemedicine), because after all, the elderly can't operate it. The elderly could be familiar with the operation through the village doctor, and he can use his knowledge points to express what he needs to go to the health service platform. This may achieve better results. If it is transmitted upward through the village doctor, and the village doctor takes the medicine, it will not cause something like wrong prescription delivery. He will check these things, which is also better for us farmers.

P6, male, 49 years

## Discussion

### Principal Findings

Understanding telemedicine use in the rural areas of China is essential for understanding the services gap and future program planning. Our study extended the existing literature by exploring the factors associated with telemedicine service use among rural residents in Guangdong Province, China. With a quantitative survey supplemented with qualitative document analysis, this study provided multiple perspectives on potential barriers to or facilitators of telemedicine use in rural China. We found that extremely few (14/2101, 0.67%) participants in the rural areas used telemedicine services. Villagers in households with family members who had fever in the past 2 weeks were more likely to use telemedicine services. In addition, smartphone or computer ownership was marginally associated with telemedicine use. The qualitative findings supported the quantitative findings. The qualitative approach mainly supplemented the results from the knowledge, trust, demand, self-efficacy, and physical and social support perspectives.

In this study, <1% (14/2101, 0.67%) of telemedicine use was found among the villagers. This finding was lower than that of a nationwide survey that included both urban and rural areas in China from 2016 to 2017, which indicated that 10% of the population used web-based health care communication or management [35]. As rural or urban residence was proven to be the most significant predictor of telemedicine use and Chinese rural residents had a distinctly lower use than urban residents [35], this finding could be explained by the disparity between rural and urban areas. Moreover, most of the respondents in rural areas were older adults, whereas most young men or labor workers moved to urban areas in China [36]. Aging has been proven to be a significant barrier to telemedicine service use [12,35]. Future programs to promote telemedicine uptake among older people would have shared value when targeting rural residents. Another important reason is that owing to chronic disease management through primary health care taking place in rural Chinese communities, which has been proven to be correlated with fewer villagers’ specialist visits and inpatient admissions [37], additional needs of the residents to seek care for these chronic diseases would be low, and generally they did not need to seek care through telemedicine.

Both quantitative and qualitative findings identified a lack of equipment (eg, smartphone or computer) or decent internet connection as barriers, which was in line with the existing findings [13,14]. Although previous studies have shown a huge mobile phone market and high network coverage among the Chinese national population [4,11], our data suggested that 67.06% (1409/2101) of smartphone or computer ownership could contribute to the underuse of telemedicine in rural areas.

Furthermore, developing fever in the past 2 weeks was associated with telemedicine service use in both quantitative and qualitative results. This could be explained by the effect of the COVID-19 pandemic outbreak on telemedicine service use. As fever is the most common symptom in patients with COVID-19, teleconsultation systems have been used to collect patient information, such as fever and cough, to control the pandemic [38]. Recent research has also found increased interest in and demand for telehealth services worldwide during the COVID-19 pandemic crisis [38]. In addition, this finding may suggest the low demand to prioritize telemedicine as a conventional health service from the patient side, for qualitative interviews described fever as a minor and common illness and mentioned physical hospitals as a prior and more reliable alternative, especially for serious or urgent conditions. Previous studies have reported available alternatives for receiving health care services [12], citing patients’ perceived low value of telemedicine or lack of motivation as barriers to public engagement with digital health services [13,14].

Lack of knowledge and skills was the most coded factor in the qualitative interviews. This is consistent with previous studies [12-14]. Our study also identified the desire for operational training support. Digital health education and training have been an important element in the process of implementing telemedicine services. Previous studies suggested limited digital literacy would increase disparities in health care access for vulnerable populations (eg, rural residents) when scaling up telemedicine implementation [39]. Without addressing this, telemedicine programs would risk excluding vulnerable groups. Similar to previous research that indicated skepticism as a barrier and self-efficacy as an enabling factor for the learning and use of eHealth technologies [14], we also found a lack of trust in telemedicine resulting from concerns about information security and quality of service and low self-efficacy of telemedicine acceptance among rural residents. This suggests additional efforts to build trust and improve self-efficacy could enhance telemedicine uptake. Furthermore, our qualitative findings also highlighted the positive impact of telemedicine practice among primary health care providers (eg, village doctors) on villagers adoption. Existing literature indicated the importance of patient-provider relationship in medication regimen adherence based on telemedicine [40], although research on the influence of telemedicine practice among primary health care providers on patients’ use is limited. Lack of accessibility and time efficiency of medication delivery services in rural areas were also identified as a barrier to telemedicine use. Research from Singapore found that besides web-based consultation, telemedicine-based medication delivery services have ramped up medical logistics supply without doctor-patient contact during the COVID-19 pandemic [41]. However, medication delivery services based on telemedicine are limited in LMICs. The practice model of remote pharmacy services relying on web-based consultation in China is still underexplored [42]; more evidence is needed to inform the development of an effective medication delivery service based on telemedicine.

### Implications for China and Other LMICs

This study had several implications for telemedicine scaling up in China and other LMICs. First, training sessions for digital literacy improvement of potential users are essential before scaling up of telemedicine services to avoid deteriorating health care inequalities, especially in rural areas of LMICs. Self-efficacy can be achieved through educational sessions. Second, similar to the findings of previous literature, lack of equipment or infrastructure has been a barrier in low-resource settings [43]; therefore, investment in rural construction could be considered as technical equipment support. However, further research on the cost-effectiveness of telemedicine program construction should be conducted. Third, our findings indicated that concerns about inadequate supervision or regulations to ensure information security and quality of web-based medical services may result in a lack of trust in telemedicine services. Thus, we recommended formulating regulations and improving the information transparency of telemedicine platforms in China and other LMICs to strengthen trust and facilitate telemedicine use. Finally, further research on establishing sustainable patient-provider relationships for telemedicine engagement and developing effective medication delivery services based on telemedicine systems is needed, especially for LMICs where related planning or regulations might not occur.

### Limitations

This study had a few limitations. First, the observational nature of the study had limitations in examining causality, but the quantitative analysis supplemented with qualitative analysis enabled us to triangulate the information to explore the correlates of telemedicine uptake in rural settings in China. Second, the proportion of people who had ever used telemedicine in quantitative analysis is very low, which limited the ability of the statistical model to detect possible associated factors such as age, gender, financial situation, smartphone or computer ownership, and geographic isolation [12-14].

### Conclusions

This study reported the profile of telemedicine use among rural patient end users in Guangdong Province, China. Less than 1% of telemedicine use was found with potential barriers, including lack of knowledge, trust, demand, low self-efficacy, and insufficient physical and social support. Our study suggested that efforts to improve digital literacy and self-efficacy, build trust, and strengthen telemedicine infrastructure support could enhance telemedicine scaling up in rural China and other LMICs.

## Appendix

Multimedia Appendix 1 Items in smart health monitoring equipment packages (July 5, 2022).

Multimedia Appendix 2 Randomization and treatments of the parent cluster randomized controlled trial (July 5, 2022).

Multimedia Appendix 3 Background characteristics of rural interviewees in Guangdong Province, China (N=27).

Multimedia Appendix 4 Interview questions based on components of the Social Cognitive Theory.

Multimedia Appendix 5 Multicollinearity diagnosis with variance inflation factor.

Multimedia Appendix 6 Tjur R2 and receiver operating characteristic of the area under the curve of multivariate analysis.

## Abbreviations

aOR: adjusted odds ratio
CRT: cluster randomized controlled trial
GDP: gross domestic product
IRB: Institutional Review Board
LMIC: low- and middle-income country
ROCAUC: receiver operating characteristic of the area under the curve
SCT: Social Cognitive Theory

## Data Availability

The data sets generated or analyzed during this study are available from the corresponding author upon reasonable request.
