# Supplementary material for: Factors Influencing Adoption and Use of Telemedicine Services in Rural Areas of China: Mixed Methods Study
Source: JMIR Public Health Surveill. 2022 Dec 23;8(12):e40771. doi: 10.2196/40771 (PMC9823570; doi:10.2196/40771)
Supplement: Multimedia Appendix 1 [file publichealth_v8i12e40771_app1.docx]

**Multimedia** **Appendix 1:**

**Items in smart health monitoring equipment packages:**

- Electronic Blood Pressure Gauge
- Routine Blood Tester (Blood Oxygen and Blood Glucose Meters)
- Infrared forehead thermometer
- Hand-operated fetal heart rate monitor
- EKG
- Routine Urine Tester
- Health Management bag
- ID card readers
- Tablets that had already downloaded the telemedicine platforms

**Images of the smart health monitoring equipment packages and their included items:**


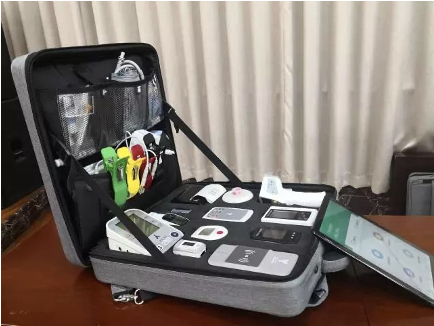
**
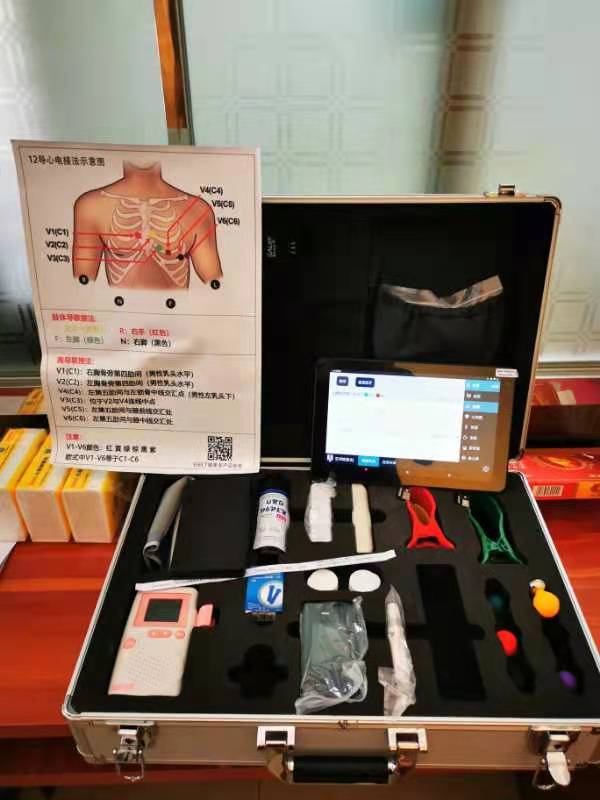
**

**
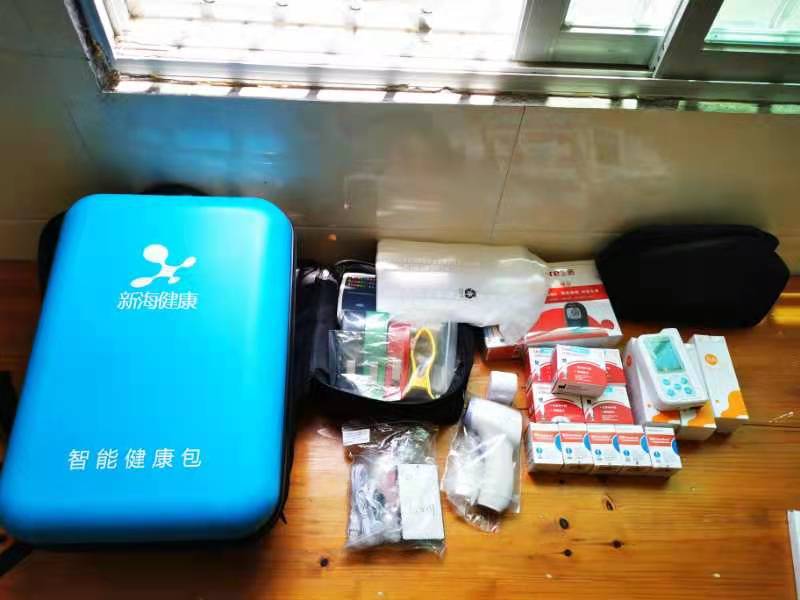
**

**Functions of the telemedicine platform:**

- **Online consultations**: Through the Dingbei Doctor portal, you can directly contact the doctors at Guangdong Second Provincial General Hospital and they will work together with the village doctors to assist with the consultation.
- **Specialist consultation and referral:** For specialized diseases, patients can contact the specialists of the Second People's Hospital of Guangdong Province for consultation and diagnosis, and treatment through the Dingbei Doctor portal. If necessary, patients can directly arrange a referral to GD2H for further diagnosis and treatment.
- **Artificial Intelligence Doctor:** The artificial intelligence doctor developed by the Second People's Hospital of Guangdong Province and Beijing Dashu Yida Technology Co., Ltd. can assist in diagnosis (self-diagnosis) and provide treatment opinions for more than 300 common diseases, and boasts a consultation accuracy rate of over 90%.

**The interface and steps of usage of the telemedicine platform:**

Fourth Step:

Through text, audio, video, etc. and various kinds of communication, specialists will complete the consultation and issue a diagnosis.

Third Step:

Click on “One-click Consultation”

Second Step:

Choose a Doctor

Online Consultation

First Step:

Click on “Online Consultation”
