# Supplementary material for: Factors Influencing Adoption and Use of Telemedicine Services in Rural Areas of China: Mixed Methods Study
Source: JMIR Public Health Surveill. 2022 Dec 23;8(12):e40771. doi: 10.2196/40771 (PMC9823570; doi:10.2196/40771)
Supplement: Multimedia Appendix 2 [file publichealth_v8i12e40771_app2.docx]

**Multimedia**  **Appendix 2:**

**Randomization of the parent cluster randomized controlled trial (CRT):**

The 144 townships were randomized across the experimental groups, stratified by the 11 total counties across the two cities. Within each township, one village was randomly selected to receive the intervention. The 144 eligible villages were randomized into five study groups in a 2:1:1:1:1 ratio, with the control group (48 villages) given twice as many villages as the other four study groups (24 villages each). Stata16.0(SE) was used for randomization.

**Treatment of each study group of the CRT:**

- **Intervention Group 1: Control (General Clinical Training Only)**

Training will be provided to one clinician from each village clinic, while doctors in this arm will not have free access to the AI doctor or telehealth throughthe Dingbei telemedicine platform. Over a 3-day course in Guangzhou and the No 2 facility and supplemental online training, the village doctors will be trained in the areas of first aid knowledge, treatment of common chronic diseases, and Chinese traditional medicine

- **Intervention Group 2: General Clinical Training + Telemedicine Platform Access and System Support**

Village clinicians will receive the same General Clinical Training as Intervention Group 1 and will additionally receive Telemedicine Platform Access and System Support. Clinicians will be given 1) a computer and mobile device loaded with the Dingbei Doctor application; 2) training on the use of the platform, and 3) ongoing technical support and service. Training on the platform will include platform introduction and field practice.

- **Intervention Group 3: General Clinical Training + Telemedicine Platform Access and System Support + Monetary Incentives:**

In addition to receiving the same General Clinical Training and Telemedicine Platform Access and System Support as the above group, clinicians in Group 3 will additionally be offered monetary incentives tied to their use of the Dingbei platform. Providers can receive a set capitation fee of 5 yuan for each verified patient visit that utilizes the telemedicine platform. To limit gaming, each village clinic randomized to an incentive group (Groups 3 and 5) will receive incentives for the first 30 valid patient visits each month. Details of the incentives will be specified in a contract between village clinicians and GD2H. It will be specified that capitation fees may only last for 3 months, after which they will be discontinued.

- **Intervention Group 4: General Clinical Training + Telemedicine Platform Access and System Support + Demand-Side Marketing**

In addition to receiving the same General Clinical Training and Telemedicine Platform Access and System Support as the above groups, a Demand Side Marketing campaign will be conducted in villages allocated to Group 4. Clinicians in this group will not receive a monetary incentive. Townships randomized into this group will also experience a social marketing campaign through three channels: banners posted at the clinic, posters/flyers placed in the village committee, and text messages which will be sent to village residents on a weekly basis.

#### **Intervention Group 5: General Clinical Training + Telemedicine Platform Access and System Support + Monetary Incentives + Demand-Side Marketing:**

Village clinicians in Group 5 will receive General Clinical Training, Telemedicine Platform Access, and System Support, and be offered Monetary Incentives tied to use of the platform as above. The Demand Side Marketing campaign will also be conducted in these villages.

- **Intervention Groups 2-5:**

Doctors randomized to groups 2-5 can receive 4 yuan for each verified patient registration to the Dingbei Doctor platform as part of the training initiative. Each village clinic in an incentive group can receive incentives for the first valid 50 patients.
