# Supplementary material for: Factors Influencing Adoption and Use of Telemedicine Services in Rural Areas of China: Mixed Methods Study
Source: JMIR Public Health Surveill. 2022 Dec 23;8(12):e40771. doi: 10.2196/40771 (PMC9823570; doi:10.2196/40771)
Supplement: Multimedia Appendix 3 [file publichealth_v8i12e40771_app3.docx]

**Multimedia**  **Appendix 3:**

**Background Characteristics of Rural Interviewees in Guangdong Province, China (N = 27)**

| No. | Age | Gender | Health needs of family members (n) | | | | Telemedicine utilization experience | Village | Interview length (minutes) |
| --- | --- | --- | --- | --- | --- | --- | --- | --- | --- |
|  |  |  | Hypertension | Diabetes | Pregnant and lying-in women | Children aged 0-6 years |  |  |  |
| P1 | 30 | Female | 0 | 1 | 0 | 0 | yes | Huangsha | 26.95 |
| P2 | 55 | Female | 0 | 0 | 0 | 2 | no | Huangsha | 28.65 |
| P3 | 60 | Male | 0 | 0 | 0 | 2 | no | Huangsha | 42.53 |
| P4 | 46 | Female | 1 | 0 | 0 | 0 | yes | Yongfu | 19.20 |
| P5 | 78 | Female | 1 | 0 | 0 | 0 | no | Yongfu | 21.87 |
| P6 | 49 | Male | 1 | 0 | 0 | 0 | no | Yongfu | 19.20 |
| P7 | 82 | Male | 0 | 1 | 0 | 0 | yes | Hankeng | 25.20 |
| P8 | 48 | Female | 1 | 0 | 0 | 0 | yes | Hankeng | 17.08 |
| P9 | 28 | Female | 0 | 0 | 0 | 1 | no | Hankeng | 17.10 |
| P10 | 55 | Male | 1 | 0 | 0 | 0 | no | Qinyang | 29.25 |
| P11 | 29 | Male | 0 | 0 | 0 | 1 | yes | Qinyang | 43.62 |
| P12 | 63 | Male | 1 | 0 | 0 | 0 | no | Qinyang | 40.97 |
| P13 | 48 | Male | 0 | 0 | 0 | 1 | no | Qinyang | 28.05 |
| P14 | 60 | Female | 2 | 2 | 0 | 0 | no | Qinglian | 13.00 |
| P15 | 72 | Male | 2 | 2 | 0 | 0 | no | Qinglian | 16.30 |
| P16 | 53 | Male | 1 | 1 | 0 | 0 | no | Qinglian | 16.07 |
| P17 | 72 | Male | 2 | 1 | 0 | 0 | no | Qinglian | 9.45 |
| P18 | 57 | Male | 0 | 1 | 0 | 1 | yes | Jingxia | 43.67 |
| P19 | 68 | Male | 0 | 1 | 0 | 0 | no | Jingxia | 35.12 |
| P20 | 25 | Female | 0 | 0 | 1 | 0 | yes | Jingxia | 28.92 |
| P21 | 49 | Female | 1 | 1 | 0 | 0 | no | Fuling* | 25.97 |
| P22 | 60 | Male | 0 | 1 | 0 | 0 | no | Fuling* | 53.92 |
| P23 | 30 | Female | 0 | 0 | 0 | 1 | yes | Fuling* | 21.67 |
| P24 | 57 | Female | 0 | 0 | 0 | 1 | no | Shuimei* | 19.87 |
| P25 | 68 | Male | 1 | 0 | 0 | 0 | no | Shuimei* | 17.72 |
| P26 | 72 | Male | 1 | 0 | 0 | 0 | no | Shuimei* | 18.02 |
| P27 | 27 | Female | 0 | 0 | 0 | 0 | yes | Shuimei* | 11.55 |

*Village selected in both quantitative and qualitative analysis.
