# Supplementary material for: Factors Influencing Adoption and Use of Telemedicine Services in Rural Areas of China: Mixed Methods Study
Source: JMIR Public Health Surveill. 2022 Dec 23;8(12):e40771. doi: 10.2196/40771 (PMC9823570; doi:10.2196/40771)
Supplement: Multimedia Appendix 4 [file publichealth_v8i12e40771_app4.docx]

**Multimedia** **Appendix 4:**

**Interview questions based on Components of Social Cognitive Theory:**

**暖场问题：**

**Warm-up Questions:**

您平时使用手机和电脑都是用来做些什么？

What do you usually use your phone and computer for?

您平时用手机微信/刷抖音/网购吗？

Do you usually use your mobile phone to browse WeChat / TikTok / online shopping?

**成分A. 个人/认知因素**

**Component A. Personal/Cognitive Factors:**

**1）知识**

**1） Knowledge**

i) 您可以说说您平时医疗和保健的相关信息的来源吗？您一般从什么地方获得例如基本医学知识，在哪里看病，买什么药/保健品的信息？

Can you talk about your usual sources of medical and health related information? Where do you usually get information such as basic medical knowledge, where to see a doctor, what medicines/health products to buy?

ii) 您听说过现在可以在网上看病吗？

Have you heard that it is now possible to see a doctor online?

- 若受访者表示没有听说过，则进行下一题的题问。

If the respondent indicated that they had not heard of it, proceed to the next question.

- 若受访者表示听说过，则需追问：

听谁说过?

是在什么场合下听说的？

If the interviewee stated that they have heard of it, make detailed inquiries:

Who have you heard from?

On what occasion did you hear it?

iii）您可以说一下**您觉得**什么是远程医疗平台吗？

Can you tell me what **you think** a telemedicine platform is?

- 如果受访者表示“不清楚”或“不知道”时则需要鼓励：

“没关系，就是说说您自己所认为的远程医疗平台是什么，想到什么就说什么，这个个问题是没有正确答案的。“

Encouragement is needed if the respondent says "unclear" or "don't know":

"It doesn't matter, just say what you think the telemedicine platform is and say whatever comes to your mind. There is no correct answer to this question.

- 若鼓励之后受访者仍表示“不清楚”/“不知道”时可以换低势能问题提示：

“提到“远程医疗平台“这个词你会想到什么？任何您觉得与他有关的人、机构、设备和过程都可以说出来，可以想到什么就说什么。“

If the respondent still expresses "unclear"/"don't know" after the encouragement, the prompt can be changed to a low potential energy question:

"What do you think of when you mention the word "Telemedicine platform"? Any person, institution, equipment and process that you think is related to him can say it, and you can say whatever you can think of."

受访者回答完毕本题后，不管回答情况如何，都需要向受访者介绍远程医疗的概念：“就是利用例如：智能手机、互联网这些信息通信技术和设备提供以诊断、治疗、预防疾病与伤害，医疗科研和评估为目的的有效信息交换的。平时遇到比较严重紧急的疾病或者需要去市里的大医院的疑难杂症就可以用用手机或者电脑上网到大城市医院的网上诊疗平台进行挂号，利用互联网找省城医院的医生看病。 “

After the respondents answer this question, no matter what the answer is, we need to introduce the concept of the telemedicine to the respondents: "It is to the delivery of healthcare services using information and communication technologies (ICT) such as smart phones, internet, for the exchange of valid information for diagnosis, treatment and prevention of disease and injuries, research and evaluation, and for the continuing education, all in the interests of advancing the health of individuals and their communities; For more serious and urgent diseases or incurable diseases that need to go to a large hospital in the city, you can use the mobile phone or computer to access the online diagnosis and treatment platform of the large city hospital for registration, and use the Internet to find a doctor in a provincial hospital for treatment.”

**2) 态度**

**2) Attitude**

i) 您觉得用远程医疗平台进行网上看病怎么样？您对网上看病有什么看法吗？

What do you think of using the telemedicine platform to see doctors online? Do you have any views on seeing doctors online?

- 如果受访者只是回答“好/不好”或者“喜欢/不喜欢“简短且笼统的评价，需要继续追问：

If the respondent just responded with a "good/bad" or "like/dislike" short and general comments, continue to ask:

为什么您觉得“好/不好”（为什么您“喜欢/不喜欢”）网上看病？可以详细说说吗？（或者您可以举个例子吗？）您觉得哪方面“好/不好”或者说您“喜欢/不喜欢”的是网上看病的哪些方面。

Why do you feel "good/bad" (why do you "like/dislike") using telemedicine? Can you elaborate? (Or can you give an example?) What do you think is "good/bad" or what you "like/dislike" about using telemedicine.

- 如果受访者回答“不知道”则需继续重复介绍问题1中互联网医院和远程医疗的概念，然继续追问他这样介绍以后觉得网上看病怎么样。如果受访者还是回答“不知道”则进行下一题提问

If the respondent answers "I don't know", we need to repeat the introduction of the concept of telemedicine in question 1, and then continue to ask him how he feels about seeing a doctor online after this introduction. If the respondent still answers," don't know", proceed to the next question

i) 您觉得网上看病对您来说有必要吗？（或者说您觉得您需要网上看病的服务吗？）

Do you think telemedicine is necessary for you? (Or do you think you need online doctor services?)

如果受访者只是回答“有必要/没有必要”则需要继续追问：

If the respondent just responded with a "yes/no" comments, continue to ask:

- 您觉得“有必要”或“没有必要”的原因是什么?

Why do you it is "necessary" or "unnecessary"?

- 您觉得什么情况下，网上看病是有必要”或“没有必要”的？您可以举个例子吗？

Under what circumstances do you think it is necessary" or "not necessary" to use telemedicine? Can you give an example?

III） 您对通过互联网提供的医疗服务有信心吗？

Do you have confidence in medical services provided over the Internet?

如果受访者只是回答“有信心/没有信心”则需要继续追问：

If the respondent just answered "yes/no" then continue to ask:

- 您觉得“有信心/没有信心”具体是指哪一方面?

可以根据以下提示词进行提示：（网上坐诊的医生能力？检查/诊断结果？网上医生提供的处方？）

What do you think "yes/no" specifically refers to?

Prompt words can be used: (Doctor ability for online consultation? Exam/diagnosis result? Prescription provided by online doctor?)

- 您信任/不信任这方面的原因是什么？

What are your reasons for trusting/distrusting this aspect?

iv）根据我们的研究，国内使用网上就医的费用大概为图文问诊均价50元，视频问诊70-100元，您觉得这个收费标准怎么样？您觉得价格会成为您使用网上就医服务的障碍吗？

According to our research, the average cost of using online medical treatment in China is about RMB 50 for image-text consultation and RMB 70-100 for video consultation. What do you think of this fee standard? Do you think price will be a barrier to your use of online medical services?

v）您认为远程医疗平台会为您提供便利还是制造不便吗？

Do you think the telemedicine platform will provide you convenience or create inconvenience?

- 如果受访者是回答“会提供便利/制造不便”则需要继续追问：

您觉得“会提供便利/制造不便”具体是指哪一方面?

If the respondent answers "will provide convenience/create inconvenience", then continue to ask:

What do you think "will provide convenience/inconvenience" specifically mean?

vi）您觉得使用远程医疗平台有什么好处和优势？和去实体的医院里看病相比有什么好处和优势？您觉得网上看病会比去实体的医院里看病有什么额外收获吗？可以详细说说您为什么会这么觉得吗？

What do you think are the benefits and advantages of using telemedicine ? What are the benefits and advantages compared to going to a physical hospital to see a doctor? Do you think seeing a doctor online will have any additional benefits over visiting a doctor in a physical hospital? Can you elaborate on why you feel this way?

vii）您觉得（如果）远程医疗服务有什么坏处和风险？和去实体的医院里看病相比有什么坏处和风险？您觉得网上看病会比去实体的医院里看病会让你有什么损失吗？可以详细说说您为什么会这么觉得吗？

What do you think are the disadvantages and risks of using telemedicine service? What are the disadvantages and risks compared to going to a physical hospital? Do you think using telemedicine will cost you anything more than going to a physical hospital? Can you elaborate on why you feel this way?

**3) 期望**

**3) Expectations**

i）您希望有人可以来教您如何使用远程医疗平台吗？可以解释一下你为什么希望/不希望/觉得无所谓吗？

（本题需追问村民“希望/不希望/无所谓”互联网医疗平台的教学工作的原因）

Do you want someone to teach you how to use the telemedicine platform? Can you explain why you want/don't want/don’t care?

(This question needs to ask villagers why they want/don't want/don't care about the teaching work of the telemedicine platform)

ii）您觉得远程医疗平台除了找医生看病还应该具备怎样的功能？

What other functions do you think the telemedicine platform should have besides finding a doctor?

iii）如果您使用远程医疗平台的话，您期望它可以对您自身及家庭成员的健康状态有怎样的改善？

对于预期的健康体提升的提示词：高血压、糖尿病等慢性病的控制/通过医学科普或在线咨询改善不良的生活方式、饮食习惯等等

If you use a telemedicine platform, how do you expect it to improve your health and that of your family members?

**成分B. 行为因素**

**Component B. Personal/Cognitive Factors:**

**1)经历/实践：**

**1) Experience/practice:**

i) 您用过远程医疗/网上看病服务吗？

Have you ever used telemedicine service？

- 如果受访者表示“没有用过”，则进行下一题的题问。
- If the respondent indicated that they have not used, proceed to the next question.
- 如果受访者表示“用过”，则需要进行以下追问：

当时用互联网医疗平台的目的是什么？

当时使用时遇到的困难是什么？

您觉得您使用过的互联网医疗平台有哪些方面需要改进？

您还记得当时使用的时候您是如何选择信任的医生来看为您病的？您会留意网站或者app上对对各位医生的评价信息吗？

If the interviewee stated that they have used it, make detailed inquiries:

What was the purpose of using the Internet medical platform?

What were the difficulties you encountered when using telemedicine?

What aspects of the telemedicine platform you have used need to be improved?

**2）自我效能：**

**2) Self-efficacy:**

i）您觉得自己有能力通过互联网的线上资源获取医疗保健相关信息吗？可以详细说说您为什么这么觉得吗？

Do you feel that you are capable of obtaining healthcare-related information through online resources on the Internet? Can you elaborate on why you feel this way?

ii）您觉得自己有能力使用相关设备（手机或电脑）进行远程医疗的操作吗？可以详细说说您为什么这么觉得吗？

Do you think you are capable of using the relevant device (mobile phone or computer) for telemedicine? Can you elaborate on why you feel this way?

iii）您觉得自己有能力与远程医疗平台上的医生沟通吗？可以详细说说您为什么这么觉得吗？

Do you feel you are capable of communicating with doctors on telemedicine platforms? Can you elaborate on why you feel this way?

iv）你觉得如果自己需要使用远程医疗平台但遇到困难的时候，自己可以找到其他人并得到他们的帮助来进行操作吗？可以详细说说您为什么这么觉得吗？

Do you think that if you need to use the telemedicine platform but encounter difficulties, you can find other people and get their help to operate it? Can you elaborate on why you feel this way?

**成分C. 环境因素**

**Component B. Environmental Factors:**

i）您觉得自己如果要使用远程医疗服务，除了自身原因之外，需要克服什么外部障碍？

In addition to personal reasons, what external barriers do you think you need to overcome if you want to use telemedicine services?

ii）如果您在村卫生室看到有村医（或者在乡镇卫生院看到有医生）使用远程医疗平台联系上级医院的医生来解决其他村民的就医需求，您会有什么想法？可以详细说说您为什么会有这种想法吗？
How do you feel if you see a village doctor in the village clinic (or see a doctor in a township health center) using a telemedicine platform to contact doctors in higher-level hospitals to solve the medical needs of other villagers? Can you elaborate on why you feel this way?

iii）如果您的家里安了无线网络，您会用互联网医疗平台看病吗？可以详细说说为什么吗？

If a wireless network is installed in your home, will you use the Internet medical platform to see a doctor? Can you elaborate on why?

iv）如果在家里或者在村诊所有家人、亲戚或者村医帮您操作的话，您会用互联网医疗平台看病吗？可以详细说说为什么吗？

If family members, relatives or village doctors help you with the operation at home or in the village clinic, would you use the Internet medical platform to see a doctor? Can you elaborate on why?

v）如果上网看病可以用医保或者社保报销，或者是远程医疗平台提供免费义诊活动，您会用互联网医疗平台看病吗？可以详细说说为什么吗？

If online medical treatment can be reimbursed by medical or social insurance, or if the telemedicine platform provides free consultation clinics, would you use the telemedicine platform to see a doctor? Can you elaborate on why?

vi）如果提供关于如何使用远程医疗平台的培训和教学活动，您会用远程医疗平台看病吗？可以详细说说为什么吗？

If training and teaching sessions on how to use the telemedicine platform are provided, would you use the telemedicine platform to see a doctor? Can you elaborate on why?

vii）如果村诊所的村医或者乡镇卫生院的医生推荐你使用远程医疗平台进行网上看病，您会用吗？可以详细说说为什么吗？

If the village doctor in the village clinic or the doctor in the township health center recommends you using the telemedicine platform for online medical treatment, would you use it? Can you elaborate on why?
