# Supplementary material for: Factors Influencing Adoption and Use of Telemedicine Services in Rural Areas of China: Mixed Methods Study
Source: JMIR Public Health Surveill. 2022 Dec 23;8(12):e40771. doi: 10.2196/40771 (PMC9823570; doi:10.2196/40771)
Supplement: Multimedia Appendix 5 [file publichealth_v8i12e40771_app5.docx]

**Multimedia Appendix 5:**

**Multicollinearity Diagnosis with Variance Inflation Factor**

| **Characteristics** | **Variance Inflation Factor (VIF)** | **Keep in model?** |
| --- | --- | --- |
| **Macro-level factor** |  |  |
| Per capital GDP of each county (yuan) | 1.07 | Yes |
| Distance from village to the town hospital (km) | 1.07 | Yes |
| Distance from village to the most frequently visited county hospital  (km) | 1.06 | Yes |
| **Household-level factor** |  |  |
| Family size (number of family members) | 1.32 | Yes |
| Financial situation (wealth index) by household asset | 1.28 | Yes |
| Family in poverty registration | 1.06 | Yes |
| **Individual-level factor** |  |  |
| Householder | 1.56 | Yes |
| Age | 1.62 | Yes |
| Gender | 1.45 | Yes |
| Education level | 1.15 | Yes |
| Having smart phone/computer | 1.36 | Yes |
| Someone can help to use the Internet in the family | 1.16 | Yes |
| **Health needs/demand** |  |  |
| Hypertension | 1.20 | Yes |
| Diabetes | 1.11 | Yes |
| Diarrhea in the past year | 1.15 | Yes |
| Cough/runny nose in the past year | 1.16 | Yes |
| Fever in the past two weeks | 1.04 | Yes |
| Unwell feeling of family members in the past two weeks (except H/D) | 1.14 | Yes |
| Frequency of the county hospital visit in the past year | 1.07 | Yes |
| Frequency of the town hospital visit in the past year | 1.09 | Yes |
| Frequency of the village clinic visit in the past year | 1.07 | Yes |
