# Supplementary material for: Factors Influencing Adoption and Use of Telemedicine Services in Rural Areas of China: Mixed Methods Study
Source: JMIR Public Health Surveill. 2022 Dec 23;8(12):e40771. doi: 10.2196/40771 (PMC9823570; doi:10.2196/40771)
Supplement: Multimedia Appendix 6 [file publichealth_v8i12e40771_app6.docx]

**Multimedia Appendix 6:**

**Tjur's R^2^ and** **ROCAUC of multivariable analysis**

| **Expanatory variable** | **Tjur's R^2 ψ^** | **ROCAUC** |
| --- | --- | --- |
| **Environmental/contextual factor** |  |  |
| Per capital GDP of each county in 2020 (yuan) | 0.116 | 0.608 |
| Distance from village to the town hospital (km) | 0.121 | 0.614 |
| Distance from village to the most frequently visited county hospital  (km) | 0.291 | 0.671 |
| Being close relative of the village doctor | 0.165 | 0.622 |
| Number of village doctors’ indoor visit in the past year | 0.353 | 0.649 |
| **Household-level factor** |  |  |
| Family size (number of family members) | 0.241 | 0.658 |
| Financial situation  (wealth index) | 1.00 | 0.792 |
| Family in poverty registration | 0.147 | 0.611 |
| **Individual-sociodemographic factor** |  |  |
| Householder | 0.120 | 0.600 |
| Age | 0.116 | 0.608 |
| Gender | 0.116 | 0.608 |
| Education level | 0.163 | 0.580 |
| Use of any medical insurance programs | 0.147 | 0.621 |
| **Access to digital health care** |  |  |
| Having smart phone/computer | 0.403 | 0.663 |
| Can be connected to the Internet | 0.044 | 0.549 |
| Someone can help to use the Internet in the family | 0.141 | 0.614 |
| **Health needs/demand of family members** |  |  |
| Hypertension | 0.251 | 0.668 |
| Diabetes | 0.235 | 0.647 |
| Diarrhea in the past year | 0.178 | 0.614 |
| Cough/runny nose in the past year | 0.157 | 0.645 |
| Fever in the past two weeks | 0.582 | 0.718 |
| Frequency of the county hospital visit in the past year | 0.189 | 0.673 |
| Frequency of the town hospital visit in the past year | 0.196 | 0.603 |
| Frequency of the village clinic visit in the past year | 0.400 | 0.695 |

**^ψ^** Tjur's R^2^  was calculated to indicate goodness-of-fit of the multivariable model

***** Receiver Operating Characteristic of the Area Under the Curve was employed to examine predictive power of multivariable model.
